# Isolation, characterization and genomic analysis of a novel lytic bacteriophage EcoPhCCP1, capable of infecting multiple strains of multidrug-resistant Escherichia coli recovered from urinary tract infections

**DOI:** 10.1099/jgv.0.002198

**Published:** 2026-02-19

**Authors:** Boris Parra, Maximiliano Sandoval, Maximiliano Matus-Köhler, Dácil Rivera, Mathias I. Hepp, Andrés Opazo-Capurro, Gerardo González-Rocha

**Affiliations:** 1 Laboratorio de Investigación en Agentes Antibacterianos (LIAA), Departamento de Microbiología, Facultad de Ciencias Biológicas, Universidad de Concepción, Concepción, Chile; 2 Grupo de Estudio en Resistencia Antimicrobiana (GRAM), Universidad de Concepción, Concepción, Chile; 3 Facultad de Medicina Veterinaria y Agronomía, Instituto de Ciencias Naturales, Universidad de Las Américas, Av. Jorge Alessandri 1160, Campus El Boldal, Concepción, Chile; 4 Centro de Vigilancia de Aguas Residuales, Centinela Biobío, Facultad de Medicina, Universidad Católica de la Santísima Concepción, Concepción, Chile

**Keywords:** antimicrobial resistance, bacteriophages, *E. coli*, wastewater

## Abstract

There is an urgent need for alternative solutions to combat multidrug-resistant (MDR) *E. coli* infections. In recent years, there has been an increase in MDR strains causing urinary tract infections (UTIs), which has resulted in more challenging treatment options, increased healthcare costs and prolonged hospital stays. The utilization of bacteriophages as a prospective modality for the management of bacterial infections has garnered significant attention. The objective of this study was to isolate and describe a phage capable of infecting MDR *E. coli* strains isolated from the urine of patients affected with UTI. The phage EcoPhCCP1 was isolated using the plaque assay from the influent of a wastewater treatment plant. The phage was characterized by phenotypic and genomic features. Morphological characteristics such as shape and size were determined using electron microscopy, and its host range was determined against multiple MDR strains. The complete genome of the phage was subjected to whole-genome sequencing and then assembled and annotated to search for virulence or antimicrobial resistance gene (ARG). VIRIDIC was employed to compare the closest phage genomes, while VICTOR and taxMyPhage were used to construct its phylogeny. EcoPhCCP1 is a tailed phage capable of infecting and propagating in multiple MDR *E. coli* strains recovered from UTI. The phage genome is 44,482 bp in length, with a GC content of 50.7 mol%, and encodes 87 ORFs, 33 of which have been previously functionally annotated. Phage EcoPhCCP1 is a *Kagunavirus*, in the recently created *Sarkviridae* family. Notably, phage EcoPhCCP1 does not harbour ARGs or virulence genes, thus rendering it a promising candidate for phage therapy against clinically significant MDR *E. coli* strains. Moreover, phage EcoPhCCP1 possesses putative anti-CRISPR proteins.

## Data Availability

The complete genome sequence of phage EcoPhCCP1 was deposited in the GenBank database of the NCBI with the accession number PQ384582.

## Introduction

*Escherichia coli* is a Gram-negative bacillus that forms part of the gut microbiota of humans and animals [1,2]. It is an opportunistic pathogen that is responsible for a wide range of infections [3,4]. *E. coli* has been identified as the primary causative agent of urinary tract infections (UTIs) on a global scale, with a propensity to manifest a wide spectrum of symptoms. In more severe cases, the infection may progress to a fatal outcome [5]. As a highly adaptable pathogen, *E. coli* poses significant treatment challenges due to a complex interplay of molecular mechanisms that enable it to evade host defences and persist [6]. It is well documented that *E. coli* strains generally exhibit antimicrobial resistance (AMR) [7,8]. According to the list of priority pathogens established by the World Health Organization [9], *Enterobacterales* resistant to third-generation cephalosporins [10] and/or carbapenems [11] are categorized as critical. AMR is a global problem that, if left unaddressed, will have disastrous consequences for future generations [1,2]. In recent years, there has been an increase in *E. coli* strains exhibiting multidrug-resistant (MDR) and extensively drug-resistant profiles [12,13], which have made treatment of UTIs more challenging, increased healthcare costs and prolonged hospital stays [14]. Consequently, there is an urgent need for the development of new effective treatments against MDR *E. coli* recovered from UTI.

The employment of bacteriophages, or bacterial viruses, to regulate bacterial populations has been mooted as a potentially efficacious approach [15,16]. It has been demonstrated that they can be utilized as a therapeutic agent in the treatment of bacterial infections in a process referred to as ‘phage therapy’ [17]. The resurgence of interest in phage therapy is attributable to several factors, including the mounting urgency to address AMR, the advancements in phage isolation, the increasing success of clinical trials and the ongoing efforts to establish regulatory frameworks. Phage therapy offers several advantages over traditional antibiotics, rendering it an attractive alternative for treating bacterial infections, especially in the face of rising antibiotic resistance. Firstly, phages are highly specific in their targeting of the bacteria responsible for infection, without causing harm to the beneficial microbiota, in contrast to the broad-spectrum activity of antibiotics, which can disrupt the gut and skin microbiomes [18,19]. Secondly, phage therapy has minimal side effects. Phages, unlike antibiotics, do not target human cells or beneficial bacteria. This characteristic results in a reduced incidence of adverse effects, such as allergies, gastrointestinal imbalances and other complications [16]. Additionally, phages are characterized by self-limiting and safe properties. Once the target bacteria have been eradicated, the phages cease replication, thereby mitigating the risk of complications arising from overuse [17]. A further advantage of phages is their capacity to penetrate biofilms, i.e. the protective barriers that bacteria form around themselves to evade the effects of antibiotics [18]. This ability to breach biofilms renders phages more efficacious in the treatment of chronic infections. Additionally, there is a reduced risk of resistance development. While bacteria can evolve to resist phages, phages can co-evolve, i.e. adapt to overcome bacterial defences, in contrast to static antibiotics [20,24]. Moreover, most of the anti-phage defence systems described in *E. coli* [25,28] have been shown to present anti-defence systems in phages [29,33]. Other advantage of phages over antibiotics is that phages represent a biologically self-limiting and biodegradable antimicrobial option that does not leave environmentally persistent chemical residues, in contrast to certain antibiotics that can accumulate in aquatic and soil ecosystems and contribute to the spread of resistance [34]. High antibiotic residues in wastewater treatment plants (WWTPs) favour the survival and growth of antimicrobial-resistant bacteria; therefore, WWTPs are ideal environments for the discovery of phages capable of infecting them [35].

Currently, few phages have been identified as effective against MDR *E. coli* strains recovered from UTI. This limited number is insufficient to prevent bacterial resistance and ensure efficacy when developing cocktails for phage therapy. Although several coliphages with therapeutic potential have been described in recent years, only a few have been reported to infect *E. coli* strains from UTIs, and their characterization in terms of host range and genomic features is usually limited. Moreover, most of the described therapeutic coliphages belong to certain families and may not represent the full diversity that could be exploited to create diverse phage cocktails for combating the emergence of resistance.

Hence, here, we describe the isolation and characterization of the novel lytic phage EcoPhCCP1, able to infect and propagate in multiple MDR *E. coli* strains recovered from UTI.

## Methods

### Bacterial strains

Forty-one *E. coli* isolates were collected from six hospitals located in the Chilean cities of Concepción, Talcahuano, Santiago, Iquique and Antofagasta between 2015 and 2022. All of them were obtained from the urine of patients affected with UTI, with no direct involvement of patients in the study (Supplementary Material 1). All isolates were confirmed as *E. coli* through standard biochemical and physiological characterization, including oxidase, indole, citrate utilization and lactose fermentation tests, as well as assessment of colony morphology and pigmentation on MacConkey and eosin methylene blue agar. In addition to * E. coli* isolates, *Salmonella enterica* ATCC 14028 and *Pseudomonas aeruginosa* ATCC 27853 were included in the host range assay as non-host Gram-negative reference strains to confirm the host specificity of phage EcoPhCCP1. All the strains were stored at −80 °C in Lysogeny Broth (LB) containing 20% glycerol and routinely cultured in LB at 37 °C.

### Sample collection and processing

Samples from a domestic WWTP in Concepción, Chile, were collected in March 2024. They consisted of 200 ml of 24-h composite water from the influent of the WWTP, prior to any treatment. The samples were kindly provided by the Centinela Biobío-UCSC Centre, a wastewater monitoring centre. Samples were collected in sterile 50-ml tubes and stored at 4 °C until use within 48 h. The samples were centrifuged at 8,000 ***g*** for 45 min at 4 °C, and the supernatant was collected to remove large particles and most bacterial cells. To remove the remaining bacterial debris while retaining the viral fraction, the supernatant was passed through a sterile 25 mm Whatman glass fibre membrane with a pore size of 0.22 µm.

### Isolation and purification of phage EcoPhCCP1

The conventional double-layer agar (DLA) method was performed [36] using 3 ml of soft agar (LB with 0.5% agar) heated at 55 °C, 100 µl of wastewater samples previously filtered and 100 µl of the overnight culture of an *E. coli* isolate obtained from UTI (UCO-452). After mixing, the solution was poured onto a plate with LB (1.5% agar) and incubated overnight at 37 °C. For purification, one lysis plaque was randomly selected and subjected to three serial DLA and isolation, placing it in 1 ml of Sodium Chloride / Magnesium sulphate (SM) buffer (MgSO_4_ • 7H_2_O 8 mM, Tris-HCl 50 mM and NaCl 100 mM). The new purified phage EcoPhCCP1 was stored at 4 °C in SM buffer.

### Propagation of phage EcoPhCCP1

The titre of the phage EcoPhCCP1 suspension was measured by DLA using multiple dilutions performed in SM buffer. Subsequently, lysis plaques were counted, and the titre was expressed as plaque-forming units per millilitre (p.f.u. ml^−1^). For the propagation of the phage, 1 ml of EcoPhCCP1 suspension at 10^5^ p.f.u. ml^−1^ was added to 100 ml of LB broth supplemented with CaCl_2_ (5 mM final concentration) and 1 ml of the overnight culture of *E. coli* UCO-452 and incubated at 37 °C overnight. The solution was centrifuged at 4,000 r.p.m. for 15 min and the precipitate with bacterial debris was discarded. The supernatant was filtered with a 0.22 µm filter and supplemented with 10% PEG8000 and 0.5 M NaCl [37] and stored overnight at 4 °C. Then, tubes were centrifuged at 8,000 r.p.m. for 2 h at 4 °C. The supernatant was discarded and the pellet with phage was resuspended in 750 µl of SM buffer. The titre of the phage suspension was then measured by DLA using multiple dilutions of the phage suspension in SM buffer, as described above.

### Morphology analysis of phage EcoPhCCP1

The morphology of EcoPhCCP1 was visualized by transmission electron microscopy using a JEOL Microscope JEM 2011 at Centro de Espectroscopía y Microscopía (CESMI) at Universidad de Concepción. For this, 50 µl of phage EcoPhCCP1 suspension was placed on glow-discharged 200 mesh copper-coated grids. Phages on the grids were incubated for 30 s before blotting off the liquid using a Whatman filter membrane. The suspensions were then fixed with 5 µl of glutaraldehyde, incubated for 10 s, and excess liquid was blotted off. The samples were stained with 3 µl of 2% uranyl acetate and incubated for 30 s. Images were analysed using ImageJ software v1.54i to calculate the length of their tail and capsid using three particles for each phage.

### Host range

The 41 *E. coli* isolated from UTI and the three ATCC strains described above were analysed to determine the host range of phage EcoPhCCP1. It was performed by DLA using 3 ml of soft agar (LB with 0.5% agar) heated at 55 °C, 100 µl of each overnight bacterial culture performed in LB broth at 37 °C and 100 µl of EcoPhCCP1 phage suspension at 10^6^ p.f.u. ml^−1^. After mixing, the solution was poured onto an LB plate (1.5% agar) and incubated overnight at 37 °C. Detection of lysis plaques indicated the capability of the phage to infect and propagate in each strain.

To determine the genetic relationship between the phage-sensitive *E. coli* strains, ERIC-PCR, or Enterobacterial Repetitive Intergenic Consensus Polymerase Chain Reaction, was used. This is a PCR-based method for genomic fingerprinting and molecular typing of bacterial strains, particularly enterobacteria such as *E. coli*. It amplifies repetitive intergenic consensus sequences to produce unique, strain-specific banding patterns, enabling the assessment of genetic diversity and clonal relationships. This technique is particularly useful as it allows for the evaluation of clonality among bacterial strains. For this purpose, the strains were grown on TSA agar overnight under aerobic conditions at 35±2 °C. Genomic DNA (gDNA) extraction was performed using Chelex 5% w/v matrix (Bio-Rad, USA). Subsequently, gDNA concentrations were adjusted to 50 ng µl^−1^. For PCR, ERIC-2 primer (5′-AAGTAAGTGACTGGGGGGGGGTGAGCG-3′) was used as previously described [38]. The amplification products were separated on a 1.5% agarose gel at 90 V for 2 h. The band patterns were analysed by GelJ v2.0 software [39] using the unweighted pair group method with arithmetic mean (UPGMA) together with the Dice-Sørensen coefficient (DICE) and a band position tolerance of 1%. Two strains were considered genetically related when they came from a node with ≥90% similarity.

### AMR of host strains

All phage-sensitive strains, along with *E. coli* ATCC 25922, were tested for carbapenemase production using Blue Carba test [40]. Antibiotic susceptibility was assessed using the disc diffusion method on Mueller–Hinton agar, according to the Clinical and Laboratory Standards Institute (CLSI) guidelines [41], against a panel of 19 antibiotics: ampicillin (AMP), ampicillin/sulbactam (SAM), ceftazidime (CAZ), cefotaxime (CTX), ceftriaxone (CRO), ceftazidime/avibactam (CZA), aztreonam (ATM), cefepime (FEP), ertapenem (ETP), meropenem (MEM), imipenem (IPM), sulfamethoxazole/trimethoprim (SXT), gentamicin (GEN), amikacin (AMK), ciprofloxacin (CIP), levofloxacin (LEV), nitrofurantoin (NIT) and fosfomycin (FOS). Tigecycline (TGC) susceptibility was determined by the minimum inhibitory concentration, using the breakpoint proposed by the US Food and Drug Administration [42], which defines susceptibility as follows for *Enterobacterales*: susceptible (S)≤2 µg ml^−1^, intermediate (I) 4 µg ml^−1^ and resistant (R)≥8 µg ml^−1^.

### Growth inhibition assay

Growth inhibition assays were performed by infecting exponentially growing *E. coli* UCO-452 cultures (OD₆₀₀ ≈ 0.2) with EcoPhCCP1 at m.o.i. of 0.01, 0.1, 1 and 10. Cultures were incubated at 37 °C with shaking, and OD₆₀₀ was recorded every 30 min for 12 h. The mean±sd of three independent experiments was calculated, and statistical significance relative to the uninfected control was determined using a two-way ANOVA.

### Efficiency of plating

The efficiency of plating (EOP) was determined by comparing the phage titre (p.f.u. ml^−1^) obtained on each susceptible host strain to that on the reference host *E. coli* UCO-452. EOP values were calculated as the ratio of p.f.u. on the test strain to p.f.u. on the reference host.

### Thermal and pH stability of phage EcoPhCCP1

For thermal stability, 5 ml of EcoPhCCP1 suspensions (10^5^ p.f.u. ml^−1^) was incubated 10 min at 25, 50, 55, 60, 65, 70 or 75 °C. Then, the titre of the phage in each tube was measured by DLA in triplicate. The temperature stability assay focused on evaluating short-term inactivation under elevated temperatures (25–75 °C). Storage stability at lower temperatures (4, −20 and −80 °C) was assessed separately, showing that EcoPhCCP1 remains viable for extended periods under these conditions (data not shown). For the pH assay, 5 ml of EcoPhCCP1 suspensions (10^5^ p.f.u. ml^−1^) were exposed to different pH values (3.0, 4.5, 7.5, 9.3 and 11.5) for 1 h at 37 ˚C. Then, the titre of the phage in each tube was measured by DLA in triplicate. For the pH sensitivity assay, a 1-h incubation period was chosen as it is a standard timeframe to simulate prolonged exposure in variable environments, such as the acidic conditions of the gastrointestinal tract. This is particularly pertinent for considering potential oral administration of phages in therapeutic contexts.

### Nucleic acid extraction of phage suspension

Prior to DNA extraction, highly concentrated phage stocks (10^11^ p.f.u. ml^−1^) were treated with DNase and RNase for the removal of genetic material belonging to bacteria. For this, 447 µl of phage suspension was mixed with 50 µl of DNase buffer, 3 µl of turbo DNase and 3 µl of RNase (5 mg ml^−1^). Subsequently, DNase was inactivated with 20 µl of EDTA (0.5 M). The solution was incubated for 5 min at room temperature and then centrifuged at 10,000 ***g*** for 90 s. The supernatant was transferred to a sterile Eppendorf tube, and DNA extraction was performed using PureLink® Viral RNA/DNA Mini Kit (Invitrogen) following the user’s manual. The purified DNA was observed on 0.8% agarose gel electrophoresis, and DNA quantification was performed via Qubit™ 4 fluorometer (Thermo Fisher Scientific).

### Sequencing and genome assembly

Sequencing was performed at SeqCenter Inc. (Pittsburgh, USA). Illumina sequencing libraries were prepared using the tagmentation and PCR-based Illumina DNA Prep kit, incorporating custom IDT 10 bp unique dual indices and targeting an insert size of 280 bp. The input DNA used for phage library preparation was 560 ng (derived from 20 µl of DNA at a concentration of 28 ng µl^−1^). No further steps for DNA fragmentation or size selection were required. Sequencing was performed on an Illumina NovaSeq X Plus system, generating 2×151 bp paired-end reads in one or more multiplexed shared-flow-cell runs.

Demultiplexing and adapter trimming were performed using BCL-convert v4.2.4 (Illumina). The quality of reads was measured using FastQC v0.12.1 tool [43]. Genome assembly was performed *de novo* using SPAdes v3.14 [44], following the recommendation of Turner *et al*. [45] using the Phage Galaxy platform [46,47]. The quality of the assembly was checked using the Quast v5.2.0 tool [48]. Initial assembly yielded multiple contigs of varied sizes with low coverage, suggesting bacterial DNA contamination and the need for subsampling. Consequently, reads were randomly subsampled following the recommendations of Shen and Millard [49] using seqtk v1.4 [50] to obtain 2% of the total reads. After a reassembly using SPAdes, we obtained a large contig with high coverage (>90) along with several shorter contigs with low coverage (<4), indicating successful assembly of a complete phage genome [49]. Contigs with low coverage, corresponding to bacterial DNA contamination, were manually removed. High-quality assembly of the EcoPhCCP1 genome was subsequently used for annotation and analysis.

### Genome analysis and annotation of phage EcoPhCCP1

A Megablast analysis [51] was performed to assess the similarity of the EcoPhCCP1 phage to other described viral genomes. Similar phages were checked on the International Committee on Taxonomy of Viruses (ICTV) webpage, and their sequences were downloaded in July 2024 from GenBank and used as references for further analysis. Pairwise comparisons of sequences were performed using the online software VIRIDIC [52]. The relationships between EcoPhCCP1 and other prokaryotic dsDNA viruses were analysed using VIPTree v4.0 [53]. For phage classification, the online services Virus Classification and Tree Building Online Resource (VICTOR) [54] and taxMyPhage [55] were used. The phylogenetic analysis was performed using a whole genome-phylogeny approach, which entails the reconstruction of evolutionary relationships based on alignments of complete genome sequences rather than individual genes or markers. Specifically, the tree was constructed by calculating average nucleotide identity (ANI) values to estimate intergenomic distances, followed by hierarchical clustering. The UPGMA algorithm was employed for clustering, utilizing an ANI-based distance matrix. This method provides a comprehensive view of genomic similarities and was implemented to classify the phage EcoPhCCP1 within its taxonomic context.

ORFs were predicted with Phanotate [56] in pharokka v1.3.2 software [57], using the PHROGs database [58], and subsequently validated manually by searches with blast v2.9.0 [51] against the NCBI non-redundant database. The tRNAs were predicted with ARAGORN v2.36 [59].

Comparison of EcoPhCCP1 phage with their closest phages was performed by aligning all annotated protein sequences using Clinker [60], and genomic map of EcoPhCCP1 phage was generated with Proksee [61].

### Detection of AMR and virulence genes in phage EcoPhCCP1

The search for genes encoding antibiotic resistance factors was performed using Resfinder 4.6.0 [62] and the resistance gene identifier in the Comprehensive Antibiotic Resistance Database (CARD) [63]. In addition, the VIRULENCEFINDER web tool [64] was employed to identify genes potentially associated with virulence factors, using a 98% identity threshold.

### Detection of Acr genes in phage EcoPhCCP1

We used diverse predictors and databases for the identification of Anti CRISPR-Cas genes in phage EcoPhCCP1, AcRanker [65], Anti-CRISPRdb [66] AcrDB [67], PaCRISPR [68] and AcrPred [69]. Some of these tools employed artificial intelligence algorithms.

## Results

**Fig. 1.**
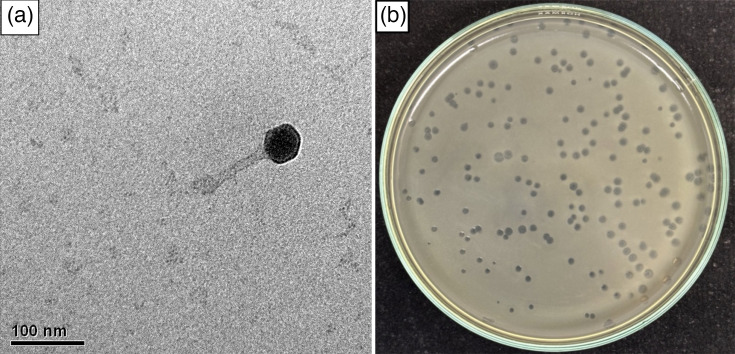
Transmission electron micrograph of the phage (**a**). Lysis plaques of phage EcoPhCCP1 on the lawn of *E. coli* UCO-452 isolate (**b**).

One lytic phage able to infect *E. coli* uropathogenic strains was isolated from wastewater samples. The isolated phage, designated as EcoPhCCP1, produced circular lysis plaques, with a diameter range of 1.7 to 2.8 mm (Fig. 1b). Morphological analysis showed that phage EcoPhCCP1 had a hexagonal capsid with an estimated diameter of 50.9±0.3 nm and a tail 139.2±4.7 nm long and 10.1±0.4 nm wide (Fig. 1a).

The host range of phage EcoPhCCP1 includes four *E. coli* pathogenic strains, UCO-452, EC-377, EC-421 and EC-425, which, according to ERIC-PCR, are not closely related (Fig. S1). EcoPhCCP1 exhibited comparable plating efficiency across all susceptible *E. coli* strains, with EOP values ranging from 0.62 to 1.0, confirming its consistent infectivity and lytic potency among genetically distinct MDR *E. coli* isolates (Fig. S2). These strains were resistant to multiple antibiotics from diverse families(Table 1). EcoPhCCP1 was unable to form plaques by DLA using strains *S. enterica* subsp. *enterica* 25928 and *P. aeruginosa* 27853.

**Table 1.** Antibiotic susceptibility profiles of *E. coli* strains sensitive to phage EcoPhCCP1

Strain	AMP	SAM	CAZ	CTX	CRO	CZA	ATM	FEP	ETP	MEM	IPM	SXT	GEN	AMK	CIP	LEV	NIT	FOS	CMI TGC
EC-377	6	6	22	6	6	30	14	8	26	30	28	6	22	24	10	15	9	29	0.125
EC-421	6	11	14	6	6	26	12	13	28	29	29	6	6	19	6	6	13	16	≤0,06
EC-425	6	18	12	16	14	36	32	24	13	32	24	6	19	19	23	24	24	25	0.125
UCO-452	6	6	6	6	6	17	32	14	16	16	17	22	19	21	32	12	22	29	≤0,06

Colours indicate the interpretation of the inhibition zone diameters according to CLSI criteria: resistant (red), intermediate (yellow), and susceptible (green). Values correspond to the diameter (in mm) of the inhibition zone for each antibiotic tested: ampicillin (AMP), ampicillin/sulbactam (SAM), ceftazidime (CAZ), cefotaxime (CTX), ceftriaxone (CRO), ceftazidime/avibactam (CZA), aztreonam (ATM), cefepime (FEP), ertapenem (ETP), meropenem (MEM), imipenem (IPM), sulfamethoxazole/trimethoprim (SXT), gentamicin (GEN), amikacin (AMK), ciprofloxacin (CIP), levofloxacin (LEV), nitrofurantoin (NIT), fosfomycin (FOS) and tigecycline (TGC).

The growth inhibition assay showed that EcoPhCCP1 effectively suppressed *E. coli* UCO-452 in a dose-dependent manner (Fig. S3). At m.o.i. 10, complete inhibition was maintained for 12 h, whereas m.o.i. 1 produced a similar inhibition pattern with only minimal recovery observed after 10 h. At lower m.o.i. (0.01–0.1), bacterial growth remained significantly reduced compared with the control but was not completely suppressed, indicating partial inhibition under these conditions. Control cultures (m.o.i. 0) displayed typical exponential growth, reaching an OD₆₀₀ of ≈ 0.6 after 12 h. The phage maintained high infectivity across the pH range 4.5–9.5, with titres of (1.8±0.2) × 10⁸ p.f.u. ml^−1^, while exposure to pH 3 and pH 11.5 resulted in reductions of ~2 and 1.5 log₁₀ units, respectively. Similarly, EcoPhCCP1 remained stable at 25–65 °C, maintaining >85% infectivity, but lost activity almost completely (>99% reduction) after incubation at 75 °C for 10 min. These results indicate that EcoPhCCP1 tolerates moderate environmental variations but is sensitive to highly acidic, alkaline or elevated thermal conditions.

### Genotypic characterization of phage EcoPhCCP1

**Fig. 2.**
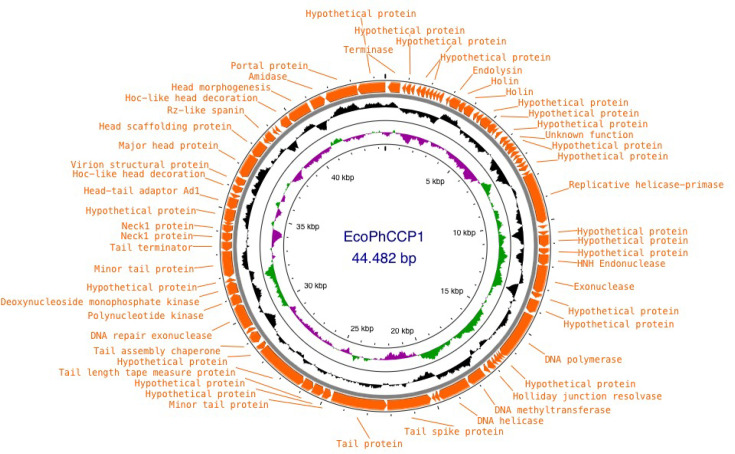
Genome map of phage EcoPhCCP1 obtained using Proksee. ORFs with identified functions and hypothetical functions are shown. Moreover, GC content and GC skew are indicated.

The genome of phage EcoPhCCP1 is a double-stranded linear DNA molecule with a length of 44,482 bp (Fig. 2). The GC content of the EcoPhCCP1 genome is 50.7%. The complete genome sequence of phage EcoPhCCP1 was deposited in the GenBank database of the NCBI with the accession number PQ384582.

Genome annotation revealed the presence of structural, replication and lysis-related genes typical of strictly lytic phages, including endolysin and holin proteins. Importantly, no genes encoding integrase, recombinase or repressor proteins were detected, confirming the absence of lysogenic potential in EcoPhCCP1.

### Phylogenetic analysis

**Fig. 3.**
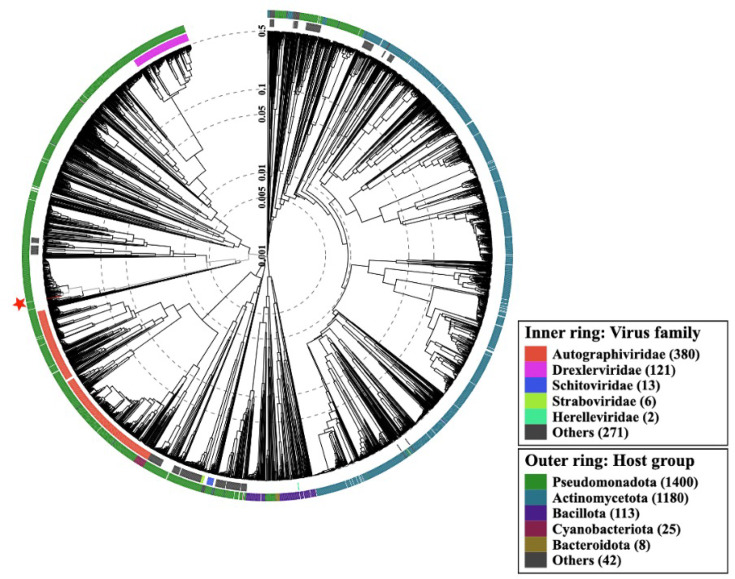
ViPTree analysis of phage EcoPhCCP1. Phages are identified according to their official ICTV classification, with the outer and inner rings indicating their host group and virus family, respectively. The asterisk marks the position of our query phage in the phylogenetic tree.

Phage EcoPhCCP1 belongs to the class *Caudoviricetes* (Fig. 3) in the recently created *Sarkviridae* family (ICTV taxonomic proposal 2024.031B). The closest relatives to EcoPhCCP1 are phages of the *Kagunavirus* genus, which have similar genome size and GC content (Table 2). The bacterial host is *E. coli* in all of them, except in phage RP180, whose bacterial host is *Raoultella* sp. [70]. However, it is probable that phage RP180 will be excluded from the genus on account of its intergenomic similarity (IS) to other members of the genus. This IS is insufficient (<70) to be considered a *Kagunavirus*.

**Table 2.** Phages related to EcoPhCCP1 belonging to the *Kagunavirus* genus

Phage	Accession	Genome size	GC content (%)	Host	Reference
EcoPhCCP1	PQ384582	44,482	50.7	*E. coli*	This work
K1ind1	NC_041897 (GU196279)	42,292	51.5	*E. coli*	[70]
Golestan	NC_042084 (MG099933)	44,829	50.5	*E. coli*	[89]
UTI-CM001	OM810255	41,222	51.0	*E. coli*	[98]
K1G	GU196277 (NC_027993)	43,587	51.0	*E. coli*	[70]
8	MZ234048	41,265	51.0	*E. coli*	[99]
K1ind2	GU196280	42,765	51.5	*E. coli*	[70]
G_AB-2017	KY295895	41,519	51.0	*E. coli*	[100,101]
L_AB-2017	KY295896	41,039	51.0	*E. coli*	[100,101]
P_AB-2017	KY295898	41,184	51.5	*E. coli*	[100,101]
RP180	NC_048181 (MK737937)	44,851	50.5	*Raoultella* sp.	[102]
K1H	GU196278 (NC_027994)	43,587	51.0	*E. coli*	[70]
ULINTec2	MZ997838	40,815	51.0	*E. coli*	[92]

**Fig. 4.**
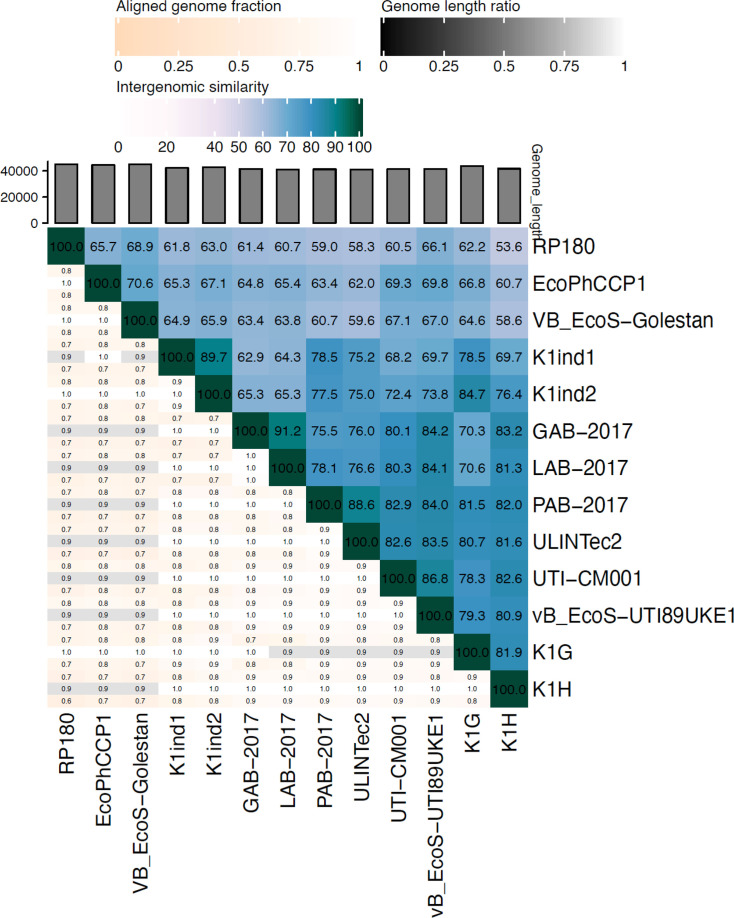
IS heatmap of EcoPhCCP1 and their related phages in the genus *Kagunavirus*.

**Fig. 5.**
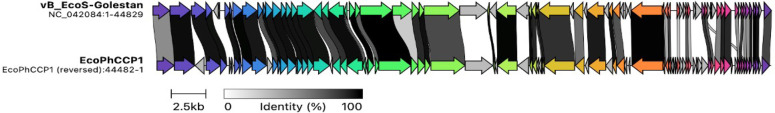
Genome comparison between EcoPhCCP1 and its closest relative Golestan using Clinker. The arrow’s colours represent the gene clusters encoding similar proteins. The lines linking the arrows show gene-encoding proteins that share more than 80% sequence identity.

VIRIDIC analysis showed the IS between EcoPhCCP1 and the members of the genus *Kagunavirus* (Fig. 4). The lower IS between EcoPhCCP1 and their relatives was observed with phage K1H, with an IS value of 60.7. On the other hand, the closest relative of phage EcoPhCCP1 is phage Golestan, with an IS of 70.6. The alignment of all annotated protein sequences between phage EcoPhCP1 and Golestan is indicated in Fig. 5.

**Fig. 6.**
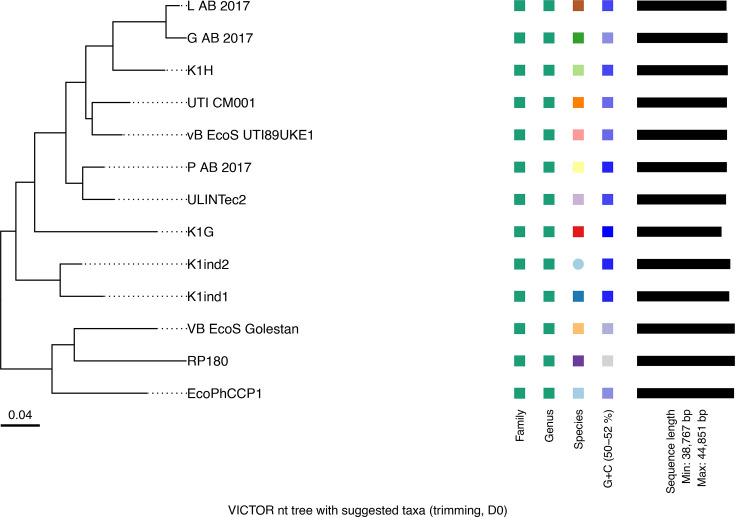
Whole genome-based phylogenetic analysis of phage EcoPhCCP1 and related Kagunavirus phages using the VICTOR method. The tree was constructed from Genome-blast Distance Phylogeny (GBDP) intergenomic distances and inferred via FASTME with SPR postprocessing. Branch support was inferred from 100 pseudo-bootstrap replicates. The scale bar indicates the intergenomic distance.

Our VICTOR analysis (Fig. 6) and taxMyPhage analysis confirm that EcoPhCCP1 is a *Kagunavirus*. Moreover, we demonstrated that all the phages classified within the *Kagunavirus* according to ICTV are distinct species. This analysis showed that our isolated phage EcoPhCCP1 is a novel species in the mentioned genus.

The identification of putative Acr proteins performed with PaCRISPR predicted a total of 13 proteins, whereas AcRAnker identified 29 proteins. However, to increase confidence in the results, we selected only those proteins that were simultaneously identified by both predictors. From this more stringent criterion, a total of eight proteins were obtained that were analysed using AcrPred. From them, the three Coding Sequences (CDSs) with a higher probability to encode a putative Acr are indicated (Table 3).

**Table 3.** Prediction of putative Acr proteins identified in EcoPhCCP1

CDS	AcrPred score	PaCRISPR score	AcRanker score	Length (aa)	Sequence
66	0.636	0.80	−2.78	57	MRECTMGTIFVIRVYDEEWDEWDIYAVGSKEEAAKEMTERSDRGQLTNFYEMPFKSE
51	0.522	0.64	−3.24	61	MKLNVGDEIYSIHSFNTFTIEYISKDGNSFVLVSSNGKYEKSRCYTLSDIKRSFKKREGKK
7	0.49	0.60	−3.24	57	MTNNEYEKLSVESATGESEIAWIDANIERYLRQVDILMERRRELINRFNLNKGDGNA

## Discussion

*E. coli* is a commensal bacterium that typically benefits its host. However, pathogenic variants of *E. coli* can significantly harm host health and frequently exhibit resistance to conventional antibiotics. As a result, bacteriophages are gaining traction as alternative treatments. In this study, a novel *Escherichia* bacteriophage EcoPhCCP1 was isolated and characterized phenotypically and genotypically.

The host range analysis of phage EcoPhCCP1 indicates that it cannot propagate in the assayed *S. enterica* and *P. aeruginosa* strains. It can infect and propagate in four *E. coli* strains: UCO-452, E-377, E-421 and E-425. Narrow host range could be considered an appreciative factor when proposing phages for phage therapy if a cocktail is used [71]. Like most viruses, phages are highly host-specific and only affect a particular bacterial species or even specific strains within that species [72,73].

The EcoPhCCP1-sensitive strains UCO-452, EC-377 and EC-421 can be categorized as MDR, because they showed non-susceptibility to at least one agent in three antimicrobial classes [74]. Moreover, these bacterial strains were isolated from patients with UTI. Strain UCO-452 is a producer of the metallo-*β*-lactamase NDM, while strains E-421 and EC-425 are producers of extended-spectrum beta-lactamases. In general, *E. coli* isolated from UTIs exhibit high resistance to beta-lactam antibiotics due to their production of beta-lactamases, making these drugs less effective for treating infections [5]. Quinolones are commonly used as first-line treatments for UTI [5,6]. Strains EC-377 and EC-421 proved to be resistant to both quinolones tested (ciprofloxacin and levofloxacin) and strain UCO-452 proved to be resistant to levofloxacin. These results confirm the relevance of having an alternative therapy to antibiotics to treat infections caused by multi-resistant bacteria.

Numerous studies have evaluated the effect of pH and temperature on bacteriophages [75,77]. Our results indicated that EcoPhCCP1 remains stable even at 65 °C, which agrees with the regular results of tailed phages. In general, these phages show stability at high temperatures, even at 80 °C [78]. However, this stability is highly dependent on the phage species [79,80]. The response of bacteriophages to different pH levels is also diverse. Studies have shown that a low pH can be detrimental to certain phages [81,83], while others show greater stability under acidic conditions [84]. EcoPhCCP1 demonstrated stability across a moderately wide pH range (4.5–9.5), comparable to other therapeutic coliphages reported in the literature. Below pH 4.0, the infectivity dropped sharply, indicating sensitivity to strong acidity. This suggests that if oral delivery were considered, protective encapsulation or buffer-based formulations would be required to ensure survival through the stomach (pH 1–4). Nonetheless, because EcoPhCCP1 targets *E. coli* strains associated with UTIs, its most relevant physiological environment is the urinary tract, where pH typically varies between 5.5 and 7.0. Within this range, the phage maintains high infectivity, supporting its potential suitability for topical, intravesical or catheter-based therapeutic applications rather than oral administration.

Phage EcoPhCCP1 is part of the *Kagunavirus* genus in subfamily *Guernseyvirinae* in the recently created family *Sarkviridae*. The genus and the subfamily were created by Anany *et al*. [85] under the proposal 2015.044fB and ratified in 2016 by the ICTV in Adams *et al*. [86]. Originally, the genus received the name *K1gvirus* and was included in the subfamily *Guernseyvirinae*, after the abolishment of the *Siphoviridae* family and the *Caudovirales* order [87]. Originally, the genus *K1gvirus* included only 4 phages, K1ind1, K1G, K1ind2 and K1H, but currently it includes 12 phages, K1ind1, K1G, K1ind2, K1H, GAB2017, Golestan, LAB2017, PAB2017, RP180, ULINTec2, UTI89UKE1 and UTICM001(Table 1). Between them, only phages UTI89UKE1, UTICM001 and Golestan have been described as able to infect and propagate in *E. coli* strains recovered from UTI.

Several other phages (accession numbers OP072527, OP030746, OP075947, OP030828, OP030929, OP031121 and BK049326) presented similar genomes to EcPhCCP1, but they are not considered as *Kagunavirus* by ICTV. All of them are metagenome assembled genomes obtained from human gut metagenomes.

According to the ICTV Subcommittee on Bacterial and Archaeal Viruses, two phages are considered the same species if they have an IS >95 and same genus if they have an IS >70 [45,88]. Our analysis showed a similarity between EcoPhCCP1 and *Kagunavirus* close to the threshold, which suggests that EcoPhCCP1 may represent a unique lineage, sufficiently divergent to establish a new genus. However, further analysis is needed to confirm that.

Golestan showed no lytic activity against *Acinetobacter baumannii*, *Staphylococcus aureus*, *P. aeruginosa, Staphylococcus epidermidis*, *Klebsiella pneumoniae*, *Klebsiella oxytoca* and *Staphylococcus saprophyticus*. However, the phage was active against 28 (53.8%) of 52 clinical *E. coli* isolates [89]. Golestan recognizes and binds to the fimbriae of *E. coli*. In this regard, it has been described that virulence factors such as fimbriae are good receptors for binding of bacteriophages, which indicates that if bacteria became resistant to phages, they can become at the same time less virulent [90]. The tail fibre proteins of EcoPhCCP1 exhibit strong sequence similarity to those of *Kagunavirus* phages Golestan and RP180, which have been associated with the recognition of fimbrial or adhesin-like receptors on *E. coli* cells. This homology suggests that EcoPhCCP1 may use similar host surface structures for attachment and infection. Identifying the specific receptor involved would be an important next step, as combining phages that target distinct receptors – such as fimbriae, outer membrane proteins or lipopolysaccharides – can enhance the effectiveness and durability of therapeutic phage cocktails.

*Kagunavirus* phages K1ind1, K1ind2, K1G, K1H and K1-ULINTec2 contain a gene coding for endosialidase, an enzyme that degrades the sialic acid present in the bacterial capsules of *E. coli* K1 (K1 capsule). EcoPhCCP1 also contains a putative protein with homology of 73% with known endosialidase protein. The bacterial capsule is also considered a virulence factor, due to it providing a protective coating for *E. coli* against phagocytosis. Bull *et al*. [70] demonstrated that the activity of endosialidase is critical for phage therapy against K1-capped *E. coli* in serum.

*Kagunavirus* phage RP180 is the unique *Kagunavirus* able to infect *Raoultella* sp. It contains a CRISPR-like Cas4 nuclease, which is one of the major proteins associated with the CRISPR systems [91]. *Kagunavirus* phage UTI-CM001 was isolated using an * E. coli* strain isolated from UTI, but no further characterization was performed. *Kagunavirus* phage ULINTec2 was isolated using * E. coli* O18:K1 (APEC 45), and according to Antoine *et al*. [92], it has a narrow host range [70]. The rest of the *Kagunavirus* phages were not characterized.

The complete description of phage EcoPhCCP1 revealed the absence of deleterious genes, such as ARGs or virulence factors. These findings suggest that EcoPhCCP1 phage could be considered a suitable candidate for use as a virulent agent against *E. coli* strains isolated from UTIs. Between the identified protein functions, we identified endolysin and holin genes of phage EcoPhCCP1. These proteins allow the lysis of the bacterial host. Endolysin function is to lyse the host cell membrane and release the phage progeny [93], while holins are responsible for opening small pores in the host membrane and permeabilize it [94]. The genomic organization of EcoPhCCP1 displays all the hallmarks of a virulent phage, with modules dedicated to DNA replication, morphogenesis and host lysis, and lacking genes associated with lysogeny. The absence of integrase or related recombination enzymes provides additional evidence that EcoPhCCP1 follows a strictly lytic replication cycle, consistent with its observed rapid lysis phenotype and the genomic architecture reported for other Kagunavirus members.

In terms of the putative Acr proteins identified in the novel phage EcoPhCCP1, to our knowledge, no anti-CRISPR proteins/genes have been searched for in any previous work on *Kagunavirus* and no Acr proteins have been described in strictly lytic *E. coli* phages. In *E. coli* mobile genetic elements, Acrs has been described and studied only in plasmids and prophages. We identified putative anti-CRISPR (Acr)-encoding genes in phage EcoPhCCP1. As previously described, Acr proteins typically lack conserved sequences, which can make their discovery challenging [95,96]. However, recent machine learning-based tools have demonstrated excellent performance in identifying such genes. Using these tools, several putative Acrs were identified in phage EcoPhCCP1; among them, we propose CDSs 66, 55 and 7 as Acr-encoding genes due to their higher combined scores. They encode for small proteins (57, 61 and 57, respectively), which is a typical size for Acrs, usually between 50 and 150 aa [97]. In addition, these genes encode hypothetical proteins without predicted function after functional annotation performed using PHANOTATE [56], a CDS-prediction tool specifically designed for phages, in Pharokka [57]. The validation of the candidate Acr-encoding genes needs experimental validation, which we will evaluate in future experiments for Acr detected in phage EcoPhPCCP1.

## Conclusion

In this study, we report the isolation of a novel bacteriophage EcoPhCCP1 from wastewater. The phage was characterized phenotypically, and its genome was thoroughly analysed. EcoPhCCP1 is a lytic dsDNA phage that belongs to the *Kagunavirus* genus. It has been shown to infect multiple MDR *E. coli* strains, associated with UTIs. Importantly, EcoPhCCP1 lacks genes related to antibiotic resistance and virulence, highlighting its potential as a biocontrol agent against this clinically relevant opportunistic pathogen. It possesses putative Anti-CRISPR systems; however, further research is needed to evaluate the functional role of the identified Acr proteins.

## Supplementary material

10.1099/jgv.0.002198 Supplementary Material 1.

## Abbreviations

AMR: antimicrobial resistance
ANI: average nucleotide identity
ARG: antimicrobial resistance gene
CDS: coding sequences
DLA: double-layer agar
EOP: efficiency of plating
gDNA: genomic DNA
ICTV: international committee on taxonomy of viruses
IS: intergenomic similarity
LB: lysogeny broth
MDR: multidrug-resistant
p.f.u. ml^−1^: plaque-forming units per millilitre
SM: sodium chloride / magnesium sulphate
UPGMA: unweighted pair group method with arithmetic mean
UTI: urinary tract infection
WWTP: wastewater treatment plant
