# Supplementary material for: Isolation, characterization and genomic analysis of a novel lytic bacteriophage EcoPhCCP1, capable of infecting multiple strains of multidrug-resistant Escherichia coli recovered from urinary tract infections
Source: J Gen Virol. 2026 Feb 19;107(2):002198. doi: 10.1099/jgv.0.002198 (PMC12919940; doi:10.1099/jgv.0.002198)
Supplement: Supplementary Material 1. [file jgv-107-02198-s001.pdf]

Supplementary material

Supplementary table 1: Characteristics of isolated *Escherichia coli* strains, including associated enzymes, source locations, sample origins, and isolation years.

E. coli strain	Enzymes	Source location	Sample origin	Year
UCO-452	NDM (+)	Clinical Hospital Universidad de Chile	Urine	2017
EC-377	x	Red Salud UC	Urine	2013
EC-421	ESBL (+)	Higueras Hospital	Urine	2022
EC-425	ESBL (+)	Higueras Hospital	Urine	2022
EC-348	x	Iquique Hospital	Urine	2009
EC-349	x	Iquique Hospital	Urine	2009
EC-350	x	Iquique Hospital	Urine	2009
EC-351	x	Iquique Hospital	Urine	2009
EC-356	x	Antofagasta Hospital	Urine	2009
EC-357	x	Puerto Montt Hospital	Urine	2009
EC-358	x	Iquique Hospital	Urine	2009
EC-359	x	Iquique Hospital	Urine	2009
EC-360	x	Iquique Hospital	Urine	2011
EC-361	x	Iquique Hospital	Urine	2011
EC-366	x	Red Salud UC	Urine	2012
EC-367	x	Red Salud UC	Urine	2012
EC-371	x	Red Salud UC	Urine	2013
EC-372	x	Red Salud UC	Urine	2013
EC-373	x	Red Salud UC	Urine	2013
EC-374	x	Concepción Regional Hospital	Urine	2013
EC-375	x	Padre Hurtado Hospital	Urine	2013
EC-376	x	San Borja Clinical Hospital	Urine	2013
EC-379	x	Red Salud UC	Urine	2013
EC-380	x	Roberto del Rio Hospital	Urine	2011
EC-409	x	Higueras Hospital	Urine	2022
EC-410	ESBL (+)	Higueras Hospital	Urine	2022
EC-411	ESBL (+)	Higueras Hospital	Urine	2022
EC-412	ESBL (+)	Higueras Hospital	Urine	2022
EC-413	ESBL (+)	Higueras Hospital	Urine	2022
EC-414	ESBL (+)	Higueras Hospital	Urine	2022
EC-415	ESBL (+)	Higueras Hospital	Urine	2022
EC-416	ESBL (+)	Higueras Hospital	Urine	2022
EC-417	ESBL (+)	Higueras Hospital	Urine	2022
EC-418	ESBL (+)	Higueras Hospital	Urine	2022
EC-419	ESBL (+)	Higueras Hospital	Urine	2022
EC-420	ESBL (+)	Higueras Hospital	Urine	2022
EC-422	ESBL (+)	Higueras Hospital	Urine	2022
EC-423	ESBL (+)	Higueras Hospital	Urine	2022
EC-424	ESBL (+)	Higueras Hospital	Urine	2022
EC-427	ESBL (+)	Higueras Hospital	Urine	2022
EC-428	ESBL (+)	Higueras Hospital	Urine	2022

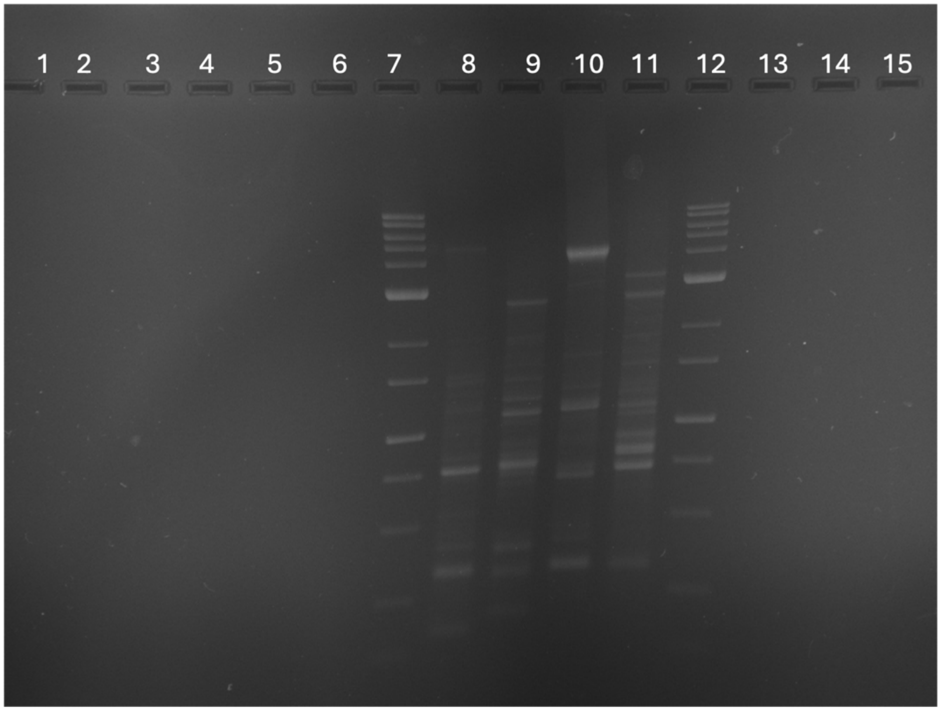

Supplementary figure 1: ERIC-PCR analysis of four clinical *Escherichia coli* isolates. Agarose gel electrophoresis (1.5%) stained with ethidium bromide showing the amplification products obtained with ERIC primers. Lanes 7 and 12 contain the 1-kb Plus DNA Ladder (molecular size marker). Lanes 8, 9, 10, and 11 contain the ERIC-PCR products of *E. coli* strains UCO-452, EC-425, EC-421, and EC-377, respectively. Distinct banding patterns indicate different genotypes and were used to assess clonal relatedness among the isolates.

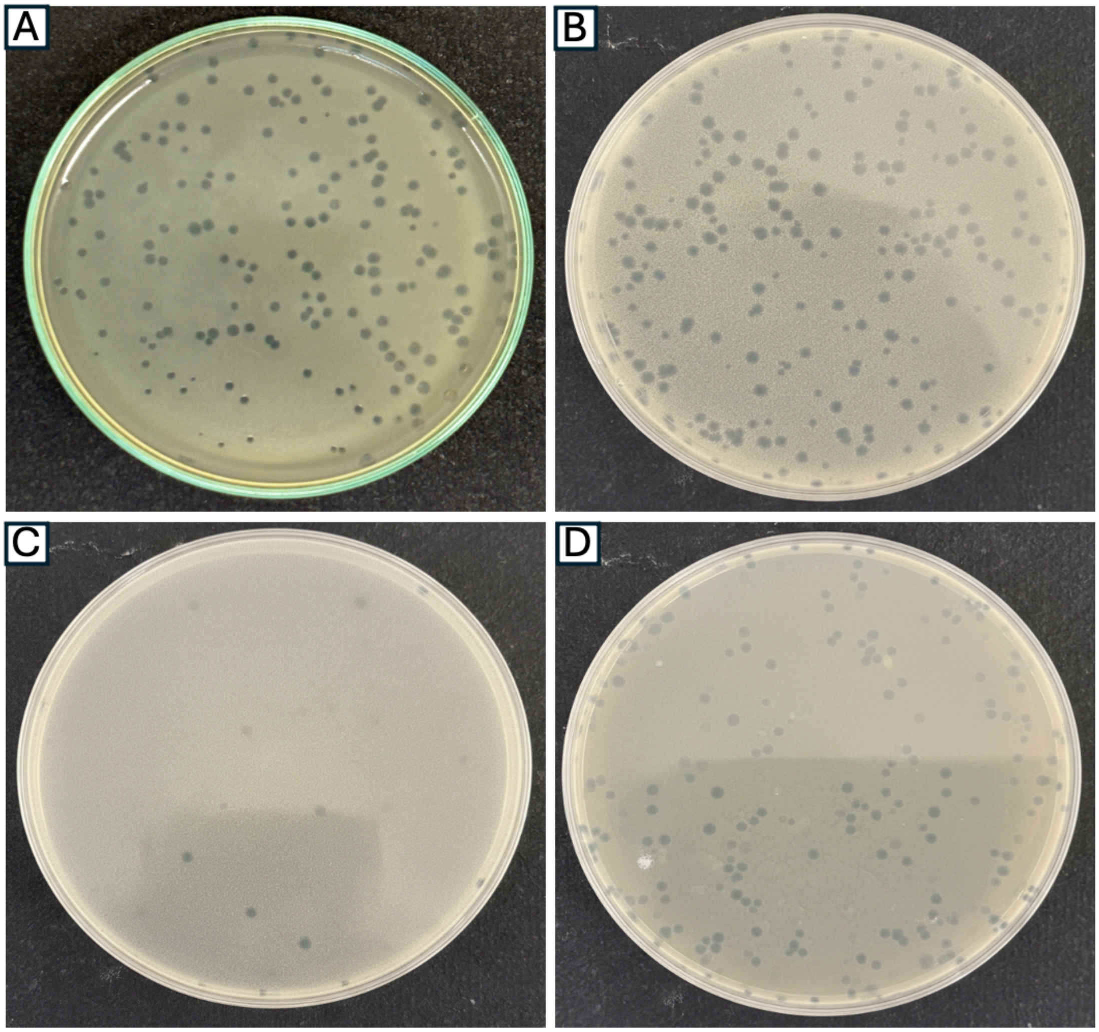

Supplementary figure 2: Spot assay results demonstrating phage susceptibility on sensitive *E. coli* strains. (A) Lysis plaques of EcoPhCCP1 phage on strain UCO-452, (B) Lysis plaques of EcoPhCCP1 phage on strain EC-377, (C) Lysis plaques of EcoPhCCP1 phage on strain EC-421 and (D) Lysis plaques of EcoPhCCP1 phage on strain EC-425.

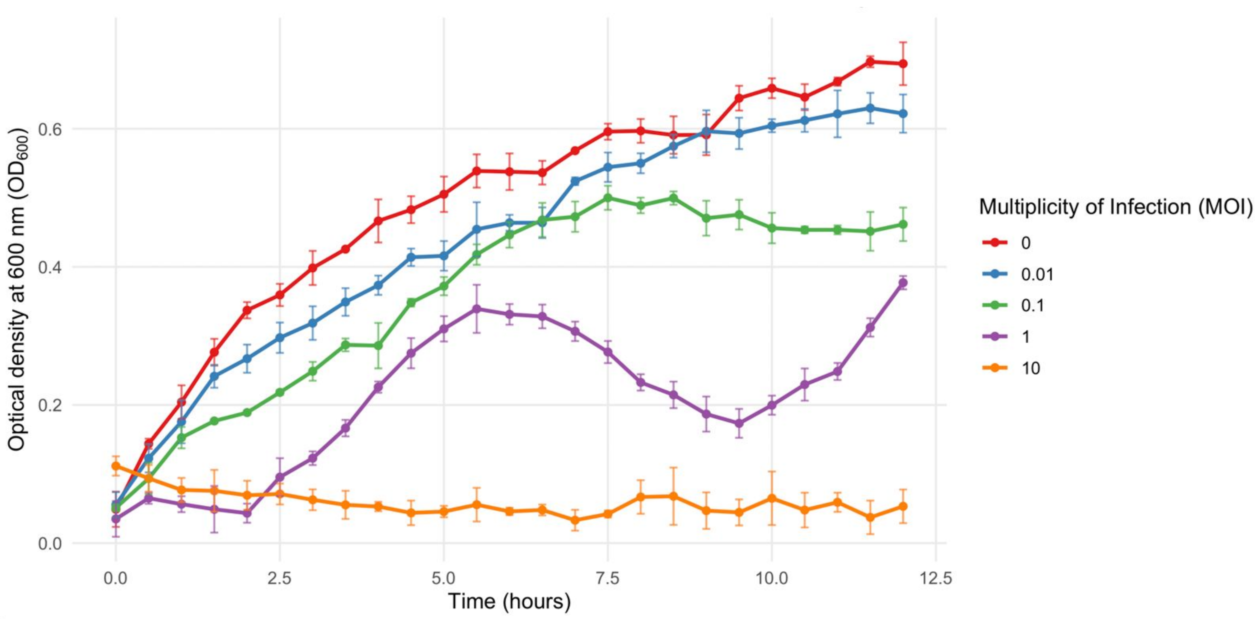

Supplementary figure 3: Growth curves of *Escherichia coli* strain UCO-452 infected with bacteriophage EcoPhCCP1 at different multiplicities of infection (MOI). Exponentially growing cultures (initial $OD_{600} \approx 0.2$) were infected at MOI 0 (uninfected control, red), 0.01 (blue), 0.1 (green), 1 (purple), and 10 (orange). Cultures were incubated at 37 °C with shaking, and OD_{600} was monitored every 30 min for 12 h. Data represent the mean ± SD of three independent experiments.
